# Supplementary material for: Evaluating the efficacy of intragastric botulinum toxin a injections with two different quantities and sites for obesity
Source: PLoS One. 2025 Dec 19;20(12):e0339259. doi: 10.1371/journal.pone.0339259 (PMC12716756; doi:10.1371/journal.pone.0339259)
Supplement: Attachment — Submitted filename: Response to Reviewers.docx [file pone.0339259.s003.docx]

Academic Editor
Professor Roland Eghoghosoa Akhigbe 
Plos One Editorial Office

Re: “Evaluating the Efficacy of Intragastric Botulinum Toxin A Injections with Two Different Quantities and Sites for Obesity “ PONE-D-24-57405

”

Dear Professor Akhigbe

We resubmitting our manuscript entitled ”Evaluating the Efficacy of Intragastric Botulinum Toxin A Injections with Two Different Quantities and Sites for Obesity”

”

The manuscript has been revised in response to the reviewers’ comments and we hope that it is now suitable for publication.

The major changes we have made are the following:

The article has been rewritten with the aid of a native English speaker. We believe that this removal of unintentional ambiguities of wording has answered many of the reviewers’ questions.

We have rewritten the method & materials, results and conclusion sections according to your comments.

We have put on the accompanying pages our responses to each of your requests, as well as our responses to the specific comments of each reviewer.

Sincerely,

Cheng-Di Chiu, MD, PhD Department of Neurosurgery, China Medical University & Hospital, Taichung, Taiwan

Email: cdchiu4046@gmail.com

**Here are our responses (by item) to the comments of reviewers. We sub-itemized the questions in order to answer them more specifically.**

Please include your full ethics statement in the ‘Methods’ section of your

manuscript file. In your statement, please include the full name of the IRB or

ethics committee who approved or waived your study, as well as whether or

not you obtained informed written or verbal consent. If consent was waived for

your study, please include this information in your statement as well.

*Ans:* We followed your suggestion and re-wrote this part in Material and Methods section **(Lines 97-101).**

Please remove or anonymize all personal information (<specific identifying information in file to be removed>), ensure that the data shared are in accordance with participant consent, and re-upload a fully anonymized data set. Please note that spreadsheet columns with personal information must be removed and not hidden as all hidden columns will appear in the published file.

*Ans:* Thank you for your notice. We have carefully reviewed the Supporting Information file and have removed all personally identifiable information, including any potentially identifying columns. All data have been anonymized in accordance with participant consent and ethical guidelines. The revised and fully anonymized dataset has been re-uploaded.

**Reviewer 1**

In the manuscript, authors stated that participants with BMI at least 25kg/M2 otherwise with complications were allowed to be enrolled. But please make it clear, whether these participants could be diagnosed with obesity or overweight or not.

*Ans:* Agreed. We re-wrote this part in **Lines 105-108 (highlighted)** to make it more clear.

Study design. Please clarify whether these participants had maintained

stable life style or weight (change less than 5%) for at least three months or

even longer at baseline or not. Also, please also clarify whether the change of

concomitant medicine or lifestyle change is allowed throughout the study.

*Ans:* Yes, we follow your suggestions and re-edit this paragraph in **Lines 134-139 (highlighted).**

Safety data was missed. Authors just simply described the efficacy data

while didn’t touch any adverse event like GI side effect which I believe is very important to evalute the treatment regimen together with efficacy data.

*Ans:* We agree with your opinion and add some descriptions on the adverse events. However, six patients sustained mild immediate post-intervention GI upset without any long-term side effects. **Lines 166-168 (highlighted).**

In the conclusion part, the authors claimed that this is a strong evidence,

which is obviously inappropriate since it is not a RCT.

*Ans:*  Agreed. We have deleted the word “ strong” in the origina**l sentence (Line 234).**

**Reviewer 2**

It is mentioned that patients were required to follow a 1200 kcal liquid diet for

12 weeks, which is quite a long time. Did patients have any difficulties with the

liquid diet? Did all patients keep their diet.

*Ans:* This diet was personalized to individual preferences, sourced from available market options, ensuring variety while maintaining nutritional balance. Therefore, all participants can keep their diet. We have re-edit this part **in Lines 134-139.**

Was there a recommended diet for patients after 12 weeks? Did you follow up with patients after changing the liquid diet? A liquid diet alone can result in significant weight loss and changing the diet after 12 weeks may lead to weight gain. A longer follow-up of patients after switching to a regular diet would be better.

*Ans:* It is very probable that in our participants a combined effect of reductive diet and toxin was observed. We agree with you. Although, all patients in this study received dietary evaluation and guidance from a dietitian both before and after the intervention, changing the diet after 12 weeks may lead to weight gain. Obesity is often closely linked to lifestyle choices. A sedentary lifestyle—characterized by minimal physical activity and prolonged sitting—combined with unhealthy eating habits, such as frequent consumption of high-calorie, processed foods, contributes significantly to weight gain and obesity.

However, our study focused on the effects of different doses and sites of intragastric BTX-A injections at this stage. We are happy to follow and help the overweight and obese patients in the long run to switch to a regular diet in our future study.

Were there any short-term or long-term complications after the procedure?

*Ans:* Six patients sustained mild immediate post-intervention GI discomforts without any long-term side effects. **Lines 166-168 (highlighted).**
