# Supplementary material for: Evaluating the efficacy of intragastric botulinum toxin a injections with two different quantities and sites for obesity
Source: PLoS One. 2025 Dec 19;20(12):e0339259. doi: 10.1371/journal.pone.0339259 (PMC12716756; doi:10.1371/journal.pone.0339259)
Supplement: Attachment — Submitted filename: Response_to_Reviewers_auresp_2.docx [file pone.0339259.s004.docx]

**Here are our responses (by item) to the comments of reviewer 2**

Thank you for your response and for addressing the comments. I believe it would be beneficial to discuss the combined effect of a reductive diet and toxins in the discussion section, as well as the importance of long-term lifestyle choices in maintaining weight.

*Ans:* We sincerely appreciate your insightful comments and constructive suggestions. Following your recommendation, we have expanded the Discussion section **(Line 217-230)** to address the combined effect of a calorie-restrictive diet and botulinum toxin A, as well as the importance of long-term lifestyle modifications in sustaining weight loss. Specifically, we emphasized that while intragastric botulinum toxin A injections may facilitate initial weight reduction, durable outcomes are contingent upon continued adherence to healthy dietary patterns and behavioral strategies over time. We also underscored that the supportive role of a calorie-restrictive, nutritionally balanced diet is likely synergistic with the pharmacological intervention, as reported in recent studies.

Additionally, if you have any considerations about the diet after three months, I would suggest including them in the manuscript.

*Ans:* Thank you for your suggestions. We incorporated remarks regarding dietary considerations beyond the three-month follow-up. Although our study period was limited to 12 weeks, we acknowledged the potential for weight regain if patients revert to pre-intervention eating habits. Accordingly, we highlighted the necessity of structured dietary counseling and lifestyle interventions to achieve and maintain long-term success.
