# Supplementary material for: Evaluating the efficacy of intragastric botulinum toxin a injections with two different quantities and sites for obesity
Source: PLoS One. 2025 Dec 19;20(12):e0339259. doi: 10.1371/journal.pone.0339259 (PMC12716756; doi:10.1371/journal.pone.0339259)
Supplement: Attachment — Submitted filename: Response_to_Reviewers_auresp_3.docx [file pone.0339259.s005.docx]

Dear Editor-in-Chief,

Enclosed is our revised manuscript entitled “Evaluating the Efficacy of Intragastric Botulinum Toxin A Injections with Two Different Quantities and Sites for Obesity.”

We sincerely appreciate the Academic Editor’s insightful comments and constructive suggestions, particularly the observation that “although the study explores different dose–site combinations of intragastric botulinum toxin A (BTX-A), it lacks a placebo or standard-of-care control group. This omission makes it impossible to isolate the pharmacological effect of BTX-A from that of the structured 1200 kcal liquid diet and repeated dietary counseling—all of which independently induce weight loss.”

We believe these revisions substantially strengthen the manuscript by situating our findings within the broader context of obesity management and by clarifying their clinical implications for achieving sustainable weight reduction.

We appreciate your time and consideration and look forward to your favorable response.

Sincerely,

Cheng-Di Chiu, MD, PhD Department of Neurosurgery, China Medical University & Hospital, Taichung, Taiwan

Address: No.2, Yu-Der Road, North Area, Taichung, Taiwan 40447, R.O.C.

Telephone: +886-4- 22052121# 5034; +886-928829256

Fax: +886-4- 22053764

Email: cdchiu4046@gmail.com

ORCID: 0000-0003-0369-0935

**Here are our responses (by item) to the comments of the academic editor**

Although the study explores different dose–site combinations of intragastric botulinum toxin A (BTX-A), it lacks a placebo or standard-of-care control group. This omission makes it impossible to isolate the pharmacological effect of BTX-A from that of the structured 1200 kcal liquid diet and repeated dietary counselling—all of which independently induce weight loss. Without a control arm: The reported reductions in body weight, BMI, and body fat could reflect dietary restriction rather than toxin efficacy. The mixed-model analysis comparing “site” and “dose” groups assumes BTX-A is effective, yet there is no baseline-to-placebo benchmark to confirm this. Prior RCTs have yielded heterogeneous results, emphasizing the need for clear differentiation between toxin and behavioral effects.

Required correction/comment: The authors must explicitly acknowledge this as a major limitation and temper the conclusion that “endoscopic intragastric BTX-A injections effectively reduce body weight, BMI, and body fat.” Alternatively, if historical or concurrent controls were available, these should be clearly described, with corresponding data or justification for comparability. Future trials should include a randomized placebo-controlled design to validate the observed effect sizes.

Response: In accordance with this recommendation, we have expanded the Discussion section (Lines 239–242) to explicitly acknowledge this limitation, emphasizing that the current study design does not permit a clear distinction between the pharmacological effects of endoscopic intragastric BTX-A injections and the concurrent influence of caloric restriction and dietary counseling.

Furthermore, we have revised the Conclusion section (Lines 251–254) to indicate that future investigations will adopt a randomized, placebo-controlled design to confirm and validate the observed effect sizes.
